# Professional Appointments

**Published:** 1852-10

**Authors:** 


### PROFESSIONAL APPOINTMENTS.

Dr. Robert E. Rogers, of the University of Virginia, has been elected to the Chair of Chemistry in the University of Pennsylvania, made vacant by the death of his brother, Dr. James B. Rogers, and is succeeded by Dr. J. Lawrence Smith, of the University of Louisiana. We congratulate the University of Virginia on having secured the services of a gentleman every way so eminently fitted for the station as Dr. Smith.

Dr. G. M. Moore, of Woodstock, Vt., takes the Chair of Anatomy in the University of Buffalo, vacated by Dr. Palmer. In the Pennsylvania Medical College, Drs. F. G. Smith, J. M. Allen, and J. J. Reese, have received appointments, and it is believed that the institution has been much strengthened by their accession to its Faculty.

